# Chemical and Sensory Characterisation of Two *Rubus rosifolius* (Red Raspberry) Varieties

**DOI:** 10.1155/2020/6879460

**Published:** 2020-06-13

**Authors:** Theresa F. Rambaran, Camille S. Bowen-Forbes

**Affiliations:** ^1^Department of Chemistry, Faculty of Science and Technology, The University of the West Indies, Mona Campus, Kingston 7, Jamaica; ^2^Institute of Applied Sciences, Faculty of Science, Technology and Environment, University of the South Pacific, Suva, Fiji

## Abstract

Raspberries are economically important fruits, being highly valued for their taste and medicinal properties. Prior to our recent finding, the occurrence of different varieties of *Rubus rosifolius* growing in Jamaica had not been previously reported. Upon close observation of the plants, differences in various physical features pointed to the existence of two distinct plant morphotypes, which were described as Red “*R*” and Wine Red “*WR*.” With an aim to determine which variety may be more favourable for value-added food production, we undertook their physicochemical and sensory analysis. This characterisation led to the rationalisation of the differences in the perceived sensory properties of these biologically active fruits. Total phenolic content was determined using the Folin-Ciocalteu reagent assay, and the identification and quantification of anthocyanins were done via HPLC-MS and HPLC-UV, respectively. Proximate and physicochemical analyses were also carried out. The findings of the analyses were associated with those of a consumer sensory analysis. The *WR* fruits had a greater quantity of the deep red anthocyanin, cyanidin-3-glucoside (66.2 mg/100 g FW), and a significantly lower lightness value. They also received a significantly higher sweetness score, which is associated with their higher total sugar content (4.8 g/100 g) and maturity index (6.7). The *R* fruits had a higher quantity of the orange-coloured pelargonidin-3-rutinoside (17.2 mg/100 g FW) and significantly higher titratable acidity (1.3 g citric acid/100 mL), the latter being associated with its significantly more sour taste. The high total phenolic contents suggest a health-functional value of these *R. rosifolius* berry fruits. Our findings, which revealed that the *WR* variety was the preferred choice among consumers, may be used to guide future product-development endeavours of these commercially valuable fruits.

## 1. Introduction

*Rubus* L. is indigenous to six continents and is one of the most diverse genera in the plant kingdom with its main constituents being raspberries and blackberries [1]. Several species of raspberries have been reported in Jamaica, and among these is the red raspberry, *Rubus rosifolius*, which is native to places such as Southeast Asia, the Caribbean, Brazil, and Hawaii [2]. The occurrence of different varieties of *R. rosifolius* grown in Jamaica was not reported prior to our recent finding. Differences in various physical features indicated the existence of two distinct plant varieties. In particular, one variety was noted to bear bright red, and the other, deeper red fruits, leading to them being respectively labelled Red “*R*” and Wine Red “*WR*.” The fruits also differed in taste, in addition to other distinctions. We therefore undertook the chemical and sensory analysis of the fruits of the two *R. rosifolius* varieties in order to characterise them and to account for the differences in their perceived sensory properties. We previously found that extracts and isolated compounds from the fruits of this species possess high antioxidant activity as well as activity against the carcinogen-activating CYP1A1 and CYP1B1 enzymes. The phenolic profiles of the fruits were also previously analysed by LC-MS. Ellagic acid, which was the major phenolic compound identified, was found to be an important contributor to the CYP1B1 activity of the methanol extracts of the two varieties [3].

A wide range of phytochemicals is present in *Rubus* berries; however, phenolics are the most abundant [4]. These fruits are particularly associated with anthocyanins, which impart their attractive red, purple, and black colours [5]. Since the difference in colour was a main point of distinction between the fruits initially, their anthocyanin profiles were examined. As polyphenols are the main cause of astringency in foods, total phenolic contents were also assessed.

The proximate profiles of the fruits were analysed and compared, as a means of characterising the fruits at a fundamental nutritional level. Their physicochemical properties were also assessed. Ripe fruits possess higher total soluble solid contents (SSC or °Brix) and pH values and lower titratable acidity (TA) than their less mature counterparts. Soluble solid contents and titratable acidity are, in fact, important contributors to fruit flavour, a particular ratio of sugar to acid being paramount for a desirable flavour. High sugar and high acid contents are required for good berry flavour, while tart/sour berries result from high acid and low sugar, and a bland taste from high sugar and low acid contents. Those possessing both low acid and low sugar contents are tasteless [6, 7]. For the sensory analysis of the *R* and *WR* raspberry varieties, the major taste descriptors assessed were sweetness, sourness, astringency, aroma, and fruity flavour. In addition to these flavour attributes, the colour of the fruit juices was also assessed.

In this study, the two Jamaican *R. rosifolius* varieties were characterised and compared by analysing their total phenolics, anthocyanin contents, proximate contents, SSC, pH, TA, and sensory characteristics. Much comparative reference was made to previous studies of *R. idaeus*, a well-known and highly commercialised species of red raspberries. With the exception of anthocyanin analysis ([8])—in which no varietal distinctions were made—to the best of our knowledge, this is the first report of these parameters in *R. rosifolius* fruits. Furthermore, this research will shed light on the variety that has greater potential for value-added food production.

## 2. Materials and Methods

### 2.1. Chemicals and Reagents

All solvents used for the extraction protocols were of Analar grade while those used for the HPLC analyses were of HPLC grade and were purchased from Sigma-Aldrich (St. Louis, MO, USA) along with all standards and reagents.

### 2.2. Fruit Samples

Ripe *R* and *WR Rubus rosifolius* fruits were collected from St. Andrew, Jamaica, from a location of elevation over 900 meters above sea level in the cool forests of the Blue and John Crow Mountains. Sample collection was done between April 2011 and January 2012. The plants were identified as *R. rosifolius* by comparison with authentic samples at the Herbarium in the Department of Life Sciences, The University of the West Indies, Mona Campus, by Patrick Lewis, the herbarium curator. Voucher specimen numbers 35595 and 35596 were assigned to the respective varieties. This batch of fruits was used for the total phenolic content determination and the identification and quantification of anthocyanins. A second batch of fruits was collected from the same location in October 2015, and these were used for all other analyses. The voucher specimen numbers assigned were 36278 and 36280 for the *R* and *WR* varieties, respectively.

### 2.3. Sample Preparation

Ripe fruits of the *R. rosifolius* varieties were kept under frozen storage at -10°C. They were then lyophilised at 200 × 10^−3^ mbar at approximately -42°C for about 4 days using a Labconco FreeZone Freeze Dryer system (Kansas City, MO, USA). The dried fruit powders of the *R* (160 g, 11.8% FW) and *WR* (202 g, 14.8% FW) fruits were taken through a series of successive extractions with n-hexane, ethyl acetate, and methanol, with a plant to solvent ratio of 1 : 4. The extraction protocol using each solvent included an initial blending at high speed for 3 mins followed by sonication of the extracts (using a Fisher Scientific Sonicator, model FS110D, MA, USA) which lasted for 1 hr. The mixture was then filtered, and the resulting filter cake was resubmerged in solvent in a similar ratio in each instance and left to percolate overnight before being filtered again. The filter cake was finally washed with the extracting solvent (150 mL) and the resulting filtrates pooled and concentrated in vacuo (using a Büchii Řotavapor R-215, Switzerland) to yield viscous yellow oils for the hexane extracts (4.68 g and 7.60 g for R_Hex_ and WR_Hex_, respectively) and reddish-brown and bright red gums, respectively, for the ethyl acetate (5.66 g: R_EtOAc_ and 6.09 g: WR_EtOAc_) and methanol extracts (79.50 g: R_MeOH_ and 98.66 g: WR_MeOH_).

### 2.4. Determination of Total Phenolic Contents

A modified method of Maurya and Singh [9] was followed, the total phenolic contents of the methanol extracts being determined using the Folin-Ciocalteu reagent. Standard solutions ranging from 1.5 to 500 *μ*g/mL gallic acid were prepared in DMSO. Plant extracts (1 mg/mL) were also prepared in DMSO, and the samples (0.5 mL) were introduced into vials and mixed with a 10-fold dilute Folin-Ciocalteu reagent (2.5 mL) and 7.5% sodium carbonate (2 mL). The vials were covered and allowed to stand for 30 mins at room temperature prior to reading at 760 nm (Thermo Scientific Helios Omega UV-Vis Spectrophotometer; Hempstead, England). All determinations were performed in triplicates and the total phenolics were expressed as mg GAE/100 g FW.

### 2.5. Qualitative Analysis of Anthocyanins

A preliminary qualitative analysis was conducted where the anthocyanin profile of the fruit extracts was assessed to identify the anthocyanins present. Fresh fruits of the *R* and *WR* plants (4 g) were macerated and sonicated for 1 hr. The resulting samples were vortexed and a small quantity (1 mL) filtered using a 0.2 *μ*m pore nylon filter cartridge. The filtrate (0.3 mL) was then passed through a C 18 Sep-Pak cartridge and the anthocyanins eluted using 0.1% HCl/MeOH (1.7 mL). The extracts were analysed by HPLC-MS (Surey HPLC system equipped with a diode array absorbance detector and a Shimadzu LCMS 2001EV system fitted with an Electrospray Interface (ESI)). A Phenomenex C18 column (4.6 mm × 250 mm) was used. Solvent A consisted of 0.1% formic acid in water and solvent B was 0.1% formic acid in methanol. The flow rate was 0.4 mL/min, and the linear gradient elution system was 95% A to 95% B over 50 mins. Positive ion mode detection (*m*/*z* M+H+) was employed. Preliminary analyses were carried out using full scan, data-dependent MS-MS scanning from *m*/*z* 250 to 1000. The interface temperature was set at 250°C, the CDL temperature at 230°C, the auxiliary gas at 1.5 L/min, and the source voltage at 1.5 kV. The anthocyanins were identified and confirmed by comparison of the spectral and chromatographic data with those previously reported by our research group (when authentic standards were used to achieve identification of the anthocyanins) [8] and with other existing literature [10].

### 2.6. Quantitative Analysis of Anthocyanins

Acidified methanol (20 mL, 0.1% HCl) was added to the lyophilised *R. rosifolius* fruits (1 g). The mixtures were then sonicated for 1 hr followed by vortexing and filtration through a 0.2 *μ*m pore nylon filter cartridge. The extraction was done in triplicates and a portion of the filtrate (1 mL) was then passed through a C18 Sep-Pak cartridge and the anthocyanins eluted with acidified methanol (2 mL). The resulting filtrate (20 *μ*L) was analysed by HPLC-UV at 520 nm using a Waters 510 HPLC system equipped with a Waters 2487 dual-wavelength absorbance detector. A Phenomenex Luna 5 *μ*m C18 column (4.6 mm × 250 mm) was employed. Standard solutions of cyanidin-3-glucoside (cy-3-glu) in 0.1% HCl/methanol were prepared by serial dilutions (12.5 to 1000 *μ*g/mL). The samples were analysed in triplicates. Solvent A consisted of 0.1% trifluoroacetic acid in water, and solvent B was 0.1% trifluoroacetic acid, 1% acetic acid, 48.5% acetonitrile, and 50.4% water. The flow rate was 0.8 mL/min, and the isocratic elution system of 73% Solvent A and 27% Solvent B was employed for 40 mins. The cy-3-glu standard was used as an external standard for the quantification of pelargonidin-3-glucoside (pel-3-glu) and pelargonidin-3-rutinoside (pel-3-rut). Additionally, the method previously used by our research group to quantify these anthocyanins using authentic standards was modelled [8].

### 2.7. Proximate Analysis

#### 2.7.1. Protein, Fat, Moisture, Ash, Total Sugar, Total Carbohydrate, and Total Calorie Contents

The protein, fat, moisture, and ash contents were analysed according to the Association of Official Agricultural Chemists official methods of analysis [11]. The nitrogen content of the lyophilised fruit samples was determined using a Kjeltec 2300 Analyzer Unit (Foss Tecator, Sweden) and protein calculated as N × 6.25. The fat contents were determined after extraction of the freeze-dried fruits with hexane using a Soxhlet extractor, and the moisture contents were analysed by drying fresh fruits at 105°C for 12 hrs using a Cole Parmer Convection Oven (IL, USA). The ash contents were determined gravimetrically after burning the dried fruits at 550°C for 12 hrs using a 48000 furnace (Thermolyne Sybron, Iowa, USA).

The reducing sugar contents of the *R* and *WR* berries were determined using the Lane-Eynon method [12]. The juices of fresh berries (40 g) from both fruit varieties were analysed in triplicates via the titrimetric method and the percentage of invert sugars in the samples was calculated as follows:

(1) $$\begin{array}{c} \%\text{Total sugar}=\frac{\text{Factor }\left(4.95\right)\times \text{dilution }\left(250\right)\times 2.5}{\text{Titre}\times \text{weight of sample }\left(\text{g}\right)\times 10}. \end{array}$$

The total carbohydrate and calorie contents were determined by use of simple mathematical calculations as outlined by FAO [13]. The carbohydrate content was determined by subtracting the total protein, fat, moisture, and ash contents present in 100 g of fresh fruits from 100. The total calorie content was determined by multiplying the percent protein and carbohydrate contents by a factor of 4 and that of the fat content by 9. The sum of the resulting values was then used to determine the total calorie contents of the fruits in kcal/100 g.

### 2.8. Physicochemical and Colourimetric Analyses

The SSC of the freshly obtained fruit juices (obtained after macerating and filtering the fresh fruits) was determined using a Pocket Refractometer (Bellingham and Stanley Ltd., Tunbridge Wells, England). The refractometer was standardised using distilled water. The TA was determined using the method of AOAC [11] Official Method 942.15 and the acidity expressed as g citric acid/100 mL juice. The pH of the fresh juices was determined using a calibrated Oakton bench-top pH meter (IL, USA).

Colourimetric analysis of the fruits was conducted using a Labscan XE colourimeter with a Universal Software V4.01 (Hunter Associates Laboratory, Reston, VA, USA). The parameters set for the instrument were mode (0/45), area view (1.75″), port size (2.00), and a UV filter (nominal). The instrument was calibrated using standard white and black reflective plates. Freshly collected fruits (3 berries) were placed in a sample holder and analysed in triplicates. The results were expressed using the L, a, b colour scale.

### 2.9. Sensory Analysis

For the sensory analysis experiments, a modified method of Vázquez-Araújo et al. [14] was followed. A total of 77 panellists—students (93%) and staff (7%) from the Faculty of Science and Technology at The University of the West Indies, Mona Campus, Kingston, Jamaica—participated in a 40-min sensory analysis session. Participants were recruited and briefed on the study and asked to complete a questionnaire. Upon satisfactory completion of the questionnaire, they were further informed of the details of the analysis and asked to read a consent form and sign the statement of declaration if interested in participating in the study. Panellists were selected once they had consumed raspberry, strawberry, cranberry, and/or pomegranate juices in the past and once their ability to recognise and perceive odours, colours, and the primary tastes was not impaired. The age distribution of the panellists was 85% between the ages of 18 and 24 years, 14% between the ages of 25 and 40 years, and 1% between the ages of 56 and 70 years. The sex distribution was 56% female and 44% male.

The sensory analysis commenced with a formal briefing to familiarise the members of the consumer panel with the concepts of astringency, sweetness, and sourness, as well as the 9-point hedonic scale. They were required to take into their mouths 20 mL of an unknown sample of fruit juice, obtained from the fresh berries, which was served at approximately 8°C. In each case, they assigned a score (1-9, with 9 representing the most intense) to the analysed juices as a representation of the intensities of sweetness, sourness, astringency, fruity flavour, aroma, and colour. The panellists neutralised the sensation and cleansed their palates between tasting each sample by rinsing their mouth with water. Panellists were required to wait for 5 mins in between samples, after which, they were asked to assess the second juice sample using the same protocol. Panellists were also asked to select the more acceptable juice overall by assigning a score to the juices (1-9, with 9 representing the most acceptable). They also rated their health perspective of the juices. The evaluation was carried out in a room with a combination of natural and fluorescent light and controlled temperature (22 ± 1°C). Ethical approval was obtained from the UWI Ethics Committee, Department of Medical Sciences, The University of the West Indies, Mona Campus, and all participants read and signed informed consent forms.

### 2.10. Statistical Analysis

A test of proportions (with normal approximation) was used to test whether subjects had a preference for one of the two samples. The characteristics of the samples were compared using Hotelling's *T*-squared generalised means test, which tests whether at least one difference is nonzero. Each characteristic was also compared separately using paired *t*-tests and confidence intervals. Differences at *p* < 0.05 were considered significant. Analyses were performed in Stata (version 15), and correlation analysis was carried out using Microsoft Excel (2016).

## 3. Results and Discussion

### 3.1. Total Phenolics and Anthocyanin Identification and Quantification

The total phenolic contents of the fruits ranged from 1.0 to 252.0 mg GA/100 g FW, depending on the extracting solvent (Table 1). The highest concentration of phenolics was obtained for the methanol extracts, R_MeOH_ and WR_MeOH_ (252 and 219 mg GA/100 g FW, respectively), followed by the ethyl acetate and hexane extracts. The difference in the phenolic contents of the respective extracts of the two raspberry varieties was however not statistically significant (*p* > 0.05). The values obtained for the methanol extracts were larger than those obtained by Dujmović Purgar et al. [15], who examined the total phenolic content of wild *R. idaeus* berries grown in Croatia, and obtained values ranging from 35.4 to 48.3 mg GAE/100 g FW, using an 80% ethanol extract. They were however lower than those reported for wild and cultivated blueberry cultivars (425-819 mg GAE/100 g FW) [16]. Vrhovsek et al. [17] recommended a daily intake of 1 g polyphenols for an average adult. Considering that approximately 73% of juice was found to result from the fresh fruits of *R* and *WR* fruits, and assuming that the content of phenolics in a neat juice sample is comparable to that of the methanol extract, a volume of approximately 300 mL of the juices (or approximately 450 g fresh berries) would satisfy the recommended daily intake (RDI), if this was the sole source of polyphenols in the diet.

A study of anthocyanins in over twenty *Rubus* species revealed only cyanidin and pelargonidin aglycones with the pelargonidins being present mostly in trace amounts [18]. In the case of *R. rosifolius* and a few other berries such as *R. pileatus*, however, pel-3-glu is the major component [8, 19]. The parent molecular ion of pel-3-glu, pel-3-rut, and cy-3-glu were found at *m*/*z* 433 (C_21_H_21_O_10_^+^), 579 (C_27_H_31_O_14_^+^), and 449 (C_21_H_21_O_11_^+^), respectively. The concentration of cy-3-glu was more than six times greater in the *WR* variety (66 mg/100 g FW), while the concentration of pel-3-rut was more than eight times greater in the R variety (17 mg/100 g FW) (Table 1). The *WR* fruits possessed a markedly greater level of total anthocyanins (219 mg/100 g FW compared to 136 mg/100 g FW in the red variety). These results differ from those obtained by Bowen-Forbes et al. [8], who assessed the anthocyanin content of *R. rosifolius* fruits (no varietal identification reported then) and obtained values of 17, 81, and 48 mg/100 g FW for cy-3-glu, pel-3-glu, and pel-3-rut, respectively. These values are, however, more closely aligned with those of the *R Rubus* variety. The estimated ratio of pelargonidins to cyanidins was greater in the *R Rubus* fruits (15 : 1) compared to the *WR* fruits (3 : 1), and these relationships also vary from those obtained by Bowen-Forbes et al. [8], who obtained a ratio of 8 : 1. Based on these data, it is likely that the sample set that was researched in that study was a mixture of both varieties, with the *R* fruits being dominant.

The total anthocyanin content of 291.7 mg/100 g FW in *R. idaeus*[20] is similar to the value obtained for the *WR* berries. The findings of the present study which show the more deeply pigmented *WR* fruits having a higher anthocyanin content support the claims of Pandey and Rizvi [21] who reported that anthocyanin content is proportional to colour intensity. Additionally, the significantly higher proportion of cyanidins compared to pelargonidins in the *WR* variety further explains its darker red colour.

### 3.2. Associations between the Instrumental Analyses and Sensory Analysis

Protein, fat, carbohydrate, moisture, ash, total sugar, and total calories were analysed in the berries, and the results are presented in Table 2. The values obtained were comparable to those reported for *R. idaeus* red raspberries [22], with the exception of the fat contents, which were notably lower in *R. rosifolius.* While the protein, fat, and ash contents of the two varieties were not significantly different (*p* > 0.05), the total sugar, total carbohydrate, and total calorie contents were significantly higher in the *WR* fruits, and the reverse was true for the moisture content (*p* < 0.05). The carbohydrate content of the *WR* raspberries (14.3%) doubles that of strawberries but is lower than that found in bananas (22.8%) [23–25]. The higher total sugar content of the *WR* fruits would suggest that these fruits are sweeter.

The SSC, pH, TA, and maturity indices (MI) of the neat juices from the two *Rubus* berries were determined (Table 2). The SSC values were found to be 6.8 and 7.0%, the *WR* fruits having the higher value; however, the values were not significantly different (*p* > 0.05). The main sugars present in the berries and, by extension, those believed to be mainly responsible for the SSC of the juices are fructose, glucose, and—to a lesser extent—sucrose [7]. The pH values of the juices were not significantly different at the 95% confidence level (*p* > 0.05); however, the TA and MI of the two fruit varieties were (*p* < 0.05). The TA was higher for the *R* fruit variety (1.3 g citric acid/100 mL juice), and the MI was greater for the *WR* fruits (6.7). According to Kader [26], there is a minimum SSC and a maximum TA content that is needed for an acceptable flavour to be perceived.

In sensorial terms, colour is thought of as a three-dimensional characteristic of appearance which consists of a lightness attribute (*L*∗), which differentiates between light and dark colours, and two chromatic parameters—*a*∗ which is the red/green coordinate and *b*∗ which is the yellow/blue coordinate. By specifying these three attributes, colours can be distinguished. The findings of the colourimetric analysis are shown in Table 2. The *L* value was greater for the *R* fruits (26). This is associated with the perceived colour of the fruits since the lightness of the colour is larger for lighter fruits. The *WR* fruits, which have a deeper colour, had a significantly smaller *L* value (20). While the fruit samples used for the anthocyanin analyses were collected 3-4 years before those for the colourimetric analyses, the colourimetric variations of the more recent batch are likely associated with the degree of differences in the anthocyanin contents of the older batches of fruits since only small variations in the latter would be expected over this duration. The findings of Miletić et al. [27] support this claim as an analysis of the anthocyanin content of plums over a 3-year period revealed that the total anthocyanin content of the fruits at peak maturity differed only by approximately 35 mg/100 mg FW. The significantly higher content of cy-3-glu (66 mg/100 g FW) (which produces a deep purple hue) is therefore likely to have contributed to the lower *L* value obtained for the *WR* fruits. The lower cy-3-glu (10 mg/100 g FW) and higher relative percentage of the pelargonidin-based anthocyanins (94%) which produce a yellow-orange colour yielded a higher *L* value in the *R* fruits, as expected.

The values obtained for lightness were statistically significant for the two varieties (*p* < 0.05), while those for the chromatic parameters (*a*∗ and *b*∗) were not. The difference in the *L* values of the two *R. rosifolius* varieties was consistent with their initial perceived colours, to which their designated names, Red “*R*” and Wine Red “*WR*,” were attributed.

**Figure 1:**
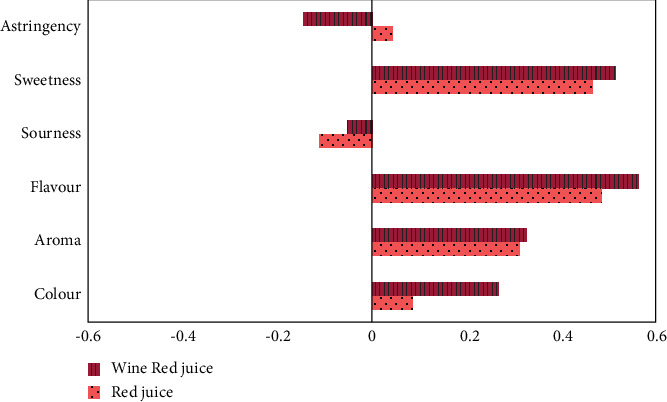
Correlations of sensory attributes with acceptability.

The consumer study was carried out to relate the objective findings of select instrumental analyses to subjective consumer perceptions and also to determine which of the juices from the two fruit varieties was considered more acceptable. The results of the sensory analysis revealed that the juice from the *WR* fruits was more acceptable (*p* < 0.001) (Table 3). This also corroborated the results of the preference test where the *WR* juice was preferred by 75% of consumers (test of proportion equals 50%: *p* < 0.001). The subjects also perceived differences in the characteristics of the two juices (Hotelling's *T*-squared generalised means test: *p* < 0.001). The fruit juices did not differ significantly in colour using the 9-point hedonic scale (*p* < 0.08). While a difference in the colour of the fresh berries was notable to the research team, this difference was not statistically significant as indicated by the panellists who assessed the resulting juices. No significant difference was detected in the aroma, flavour, and astringency of the two fruit juices. Significant differences were however obtained for sourness and sweetness. The scores for sourness from both juices (6.6 and 5.9 for the *R* and *WR* fruits, respectively) were the highest scores received among the taste parameters, an indication of the taste attribute perceived to be the most dominant in the fruits. The juice from the *R* berries was perceived as being more sour while that of the *WR* variety was deemed to be sweeter. This higher sweetness partly explains its higher acceptability. A correlation of all the subjective sensory parameters with the perceived acceptability of the juices is shown in Figure 1. Sweetness and flavour had the greatest correlations to acceptability (moderate) while a negative correlation was found for sourness for both juices. Astringency was also negatively correlated with the acceptability of the *WR* juice.

The sourness of the juices is strongly associated with both their TA and pH. Likewise, astringency is strongly associated with phenolic contents. The perceived sweetness, on the other hand, is associated with the total sugar contents and MI of the juices. The acceptability of the *WR* juice appears to be linked to its higher sweetness, total sugar contents, and MI.

Fifty percent of the panellists declared that the main factor that influences their purchase of berry juices is the associated health benefits. The other main factors influencing purchase were the taste of the juices and the need to quench thirst, which accounted for 23% and 12%, respectively. The majority of the panellists (90%) indicated that they view berry juices as healthy juice options. Other panellists (3%) declared that the juices are viewed as both a healthy option and a symbol of status, while the remaining panellists (7%) indicated that they view berry juices as any other drink. Overall, 50% of the consumers indicated that they are aware of the health benefits associated with the consumption of berry juices. The main benefit noted (by 67% of the consumers) was the antioxidant potential of the phytochemicals present within the fruits. Among the other benefits mentioned were cancer fighting potential and protection from heart disease.

## 4. Conclusions

From the present study, valuable information on the characterisation of two recently distinguished *R* and *WR* varieties of *Rubus rosifolius* berries was unveiled. Our findings indicate that significant phytochemical and physicochemical differences exist between the two varieties. While the total phenolic contents were not significantly different, the levels of cy-3-glu and pel-3-glu were significantly greater in the *WR* fruits, while pel-3-rut was more dominant in the *R* fruit variety. Several phytochemical constituents and physicochemical parameters appear to have strong association with the perceived sensory attributes of the fruits. Associations were observed between the perceived sourness and the TA and pH of the juices, the *R* fruit (which had significantly greater TA values) being deemed more sour. Sweetness was strongly associated with the total sugar content and MI of the fruits, the *WR* variety (which had significantly greater values for both parameters) being identified as sweeter. The *WR* fruits, which were significantly sweeter and less sour, were the variety preferred by the consumer panel. The impact of these two parameters on acceptability was further corroborated by means of a correlation analysis which revealed that the acceptability of the juices was positively and negatively correlated with sweetness and sourness, respectively. These results indicate that the Wine Red “*WR*” variety may be the more favourable option for the development of value-added food products.

## Figures and Tables

**Table 1:** Total phenolics and anthocyanin contents of fruit extracts from the Red “*R*” and Wine Red “*WR*” *Rubus rosifolius* varieties.

Parameters		Red “*R*”	Wine red “*WR*”
Total phenolic contents (mg GA/100 g FW)	Hexane extract	0.9 ± 0.3^a^	2.0 ± 0.4^a^
	Ethyl acetate extract	8.1 ± 0.1^a^	8.2 ± 1.0^a^
	Methanol extract	252.4 ± 20.9^a^	219.1 ± 12.7^a^
Anthocyanin contents (mg/100 g FW)	Cy-3-glu	10.4 ± 1.3^a^	66.2 ± 8.2^b^
	Pel-3-glu	135.8 ± 6.2^a^	219.0 ± 3.3^b^
	Pel-3-rut	17.2 ± 0.1^a^	2.1 ± 0.4^b^
	Total anthocyanin content^∗^	163.4 ± 7.5^a^	287.4 ± 4.2^b^

Values are the mean ± SD (*n* = 3). Different superscript letters across a row indicate that the value is significantly different at the 95% confidence level. ^∗^Values expressed as cyanidin-3-glucoside equivalents.

**Table 2:** Proximate, physicochemical, and colourimetric analyses of the Red “*R*” and Wine Red “*WR*” *Rubus rosifolius* varieties.

Parameters		Red “*R*”	Wine red “*WR*”
Proximate analysis (value/100 g FW)	Moisture (g)	87.7 ± 0.2^a^	83.4 ± 0.5^b^
	Protein (g)	1.6 ± 0.2^a^	1.8 ± 0.3^a^
	Fat (g)	0.2 ± 0.0^a^	0.1 ± 0.0^a^
	Ash (g)	0.4 ± 0.0^a^	0.4 ± 0.0^a^
	Total sugar (g)	3.6 ± 0.0^a^	4.8 ± 0.0^b^
	Total carbohydrate (g)	10.1 ± 0.5^a^	14.3 ± 0.8^b^
	Total calorie (kcal)	48.1 ± 2.9^a^	65.3 ± 4.3^b^
Physicochemical analysis	°Brix	6.8 ± 0.1^a^	7.0 ± 0.1^a^
	pH	2.9 ± 0.0^a^	3.0 ± 0.0^a^
	TA (g/100 mL)	1.3 ± 0.0^a^	1.1 ± 0.0^b^
	MI	5.4 ± 0.2^a^	6.7 ± 0.1^b^
Colourimetric analysis	*L*∗	26^a^	20^b^
	*a*∗	31^a^	31^a^
	*b*∗	21^a^	18^a^

TA: titratable acidity; MI: maturity index; *L*∗: lightness; *a*∗: red/green coordinate; *b*∗: yellow/blue coordinate. Values are the mean ± SD (*n* = 3). Different superscript letters across a row indicate that the value is significantly different at the 95% confidence level (*p* < 0.05).

**Table 3:** Consumer sensory analysis of the two *Rubus rosifolius* varieties *WR* and *R.*

Sensory attribute	Wine red “*WR*”		Red “*R*”		Difference		
	*N*	Mean (SD)	*N*	Mean (SD)	*N*	Difference in means (95% CI^∗^)	*p* value^†^
Colour	77	6.4 (1.4)	77	6.8 (1.2)	77	-0.5 (-0.9, 0.0)	0.08
Aroma	77	5.7 (1.8)	77	5.4 (2.0)	77	0.3 (-0.4, 1.0)	1.00
Flavour	77	5.4 (2.0)	77	5.0 (2.2)	77	0.4 (-0.2, 1.0)	0.41
Sourness	77	5.9 (2.0)	76	6.6 (1.7)	76	-0.7 (-1.4, -0.1)	0.03
Sweetness	77	3.5 (1.7)	77	2.8 (1.5)	77	0.7 (0.3, 1.2)	<0.001
Astringency	76	5.3 (2.2)	77	5.4 (2.4)	76	-0.0 (-0.7, 0.7)	1.00
Acceptability	77	5.0 (2.3)	77	4.0 (1.9)	77	1.0 (0.4, 1.6)	<0.001

Abbreviations: SD: standard deviation; CI: confidence interval. ^∗^Paired *t* confidence intervals, adjusted for 7 comparisons using the Bonferroni method. ^†^Paired *t*-test, adjusted for 7 comparisons using the Bonferroni method.

## Data Availability

All relevant data has been provided in the manuscript. Should any additional details be required, we will gladly provide these.

## Additional Points

*Research Highlights*. (1) Chemical and sensory properties of *R. rosifolius* varieties grown in Jamaica were determined for the first time. (2) Significant differences in a number of chemical and sensory properties were observed between the fruit varieties. (3) The Wine Red *R. rosifolius* variety “*WR*” was preferred over the Red variety “*R*” by consumers. (4) This study will inform future product-development endeavours of these commercially valuable fruits.
